# Anti-double Stranded DNA Antibodies: Origin, Pathogenicity, and Targeted Therapies

**DOI:** 10.3389/fimmu.2019.01667

**Published:** 2019-07-17

**Authors:** Xiaoyu Wang, Yumin Xia

**Affiliations:** Department of Dermatology, The Second Affiliated Hospital, School of Medicine, Xi'an Jiaotong University, Xi'an, China

**Keywords:** anti-dsDNA antibody, catalysis, lupus nephritis, peptide, suppressor of cytokine signaling 1 (SOCS1), systemic lupus erythematosus (SLE)

## Abstract

Systemic lupus erythematosus (SLE) is characterized by high-titer serological autoantibodies, including antibodies that bind to double-stranded DNA (dsDNA). The origin, specificity, and pathogenicity of anti-dsDNA antibodies have been studied from a wider perspective. These autoantibodies have been suggested to contribute to multiple end-organ injuries, especially to lupus nephritis, in patients with SLE. Moreover, serum levels of anti-DNA antibodies fluctuate with disease activity in patients with SLE. By directly binding to self-antigens or indirectly forming immune complexes, anti-dsDNA antibodies can accumulate in the glomerular and tubular basement membrane. These autoantibodies can also trigger the complement cascade, penetrate into living cells, modulate gene expression, and even induce profibrotic phenotypes of renal cells. In addition, the expression of suppressor of cytokine signaling 1 is reduced by anti-DNA antibodies simultaneously with upregulation of profibrotic genes. Anti-dsDNA antibodies may even participate in the pathogenesis of SLE by catalyzing hydrolysis of certain DNA molecules or peptides in cells. Recently, anti-dsDNA antibodies have been explored in greater depth as a therapeutic target in the management of SLE. A substantial amount of data indicates that blockade of pathogenic anti-dsDNA antibodies can prevent or even reverse organ damage in murine models of SLE. This review focuses on the recent research advances regarding the origin, specificity, classification, and pathogenicity of anti-dsDNA antibodies and highlights the emerging therapies associated with them.

## Introduction

Systemic lupus erythematosus (SLE) is a severe disease that involves dysregulation of the immune system, excessive production of pathogenic autoantibodies (and their upregulation in serum), and multiple immune-system-mediated injuries. Anti-double-stranded (ds)DNA antibodies have been some of the classic diagnostic and nosological criteria of SLE since 1982. In 2012, the high titer of anti-dsDNA antibodies in serum accompanied by biopsy-proven lupus nephritis (LN) was accepted as an independent classification criterion for SLE by the Systemic Lupus International Collaborating Clinics (1). However, the wide spectrum of molecular antibody specificity and complex contexts of antibody generation, as well as the diverse antigen structures to which these antibodies bind, make anti-dsDNA antibodies difficult to accept without further distinction as a classification criterion for SLE (1). Pathogenic anti-dsDNA autoantibodies react with DNA but are not strictly specific to it. Multiple self-antigens can be recognized by anti-dsDNA antibodies, subsequently triggering apoptosis, inflammatory responses, and tissue fibrosis. Recently, synthesized peptides that mimic a molecular DNA structure were reported to specifically recognize and interact with the anti-dsDNA antibodies, thus pointing to a novel therapeutic opportunity via inhibition of the antigen recognition of anti-dsDNA antibodies (2, 3). This review sheds light on the involvement of anti-dsDNA antibodies in the progression of SLE and provides new insights into the therapies for SLE.

## The Origin of Anti-dsDNA Antibodies

Normally, nuclear antigens, such as dsDNA, are not accessible to the immune system because they are restricted to the nucleus and mitochondria and are quickly degraded by DNases in the cytoplasm and endosomes. However, nuclear materials can be released from apoptotic cells after exposure to ultraviolet light, infection, and drugs. Cells undergo death via different processes. During NETosis, cells extrude DNA and neutrophil extracellular traps (NETs), in which DNA is covered by anti-bacterial substances (4, 5). In apoptosis, DNA cleavage leads to the formation of apoptotic bodies, which contain DNA-bound microparticles (6). In necrosis, high-molecular-weight DNA is liberated after cell lysis.

The released DNA can be recognized by anti-DNA antibodies and compose immune complexes. Extracellular nucleic acids containing immune complexes can be captured by immature dendritic cells (iDCs) via FcγRs; these cells subsequently get activated and migrate from peripheral tissues to lymphatic organs, where iDCs undergo maturation (7). Activated DCs induce the over-representation of costimulatory molecules, such as CD80, CD86, and PD-L1 (8, 9). Furthermore, these DCs trigger redistribution of the MHC II molecule and production of proinflammatory cytokines, such as interferon α (IFN-α), tumor necrosis factor (TNF)-α, and interleukin 6 (IL-6), which are inter-related with the activation of B cells and T cells (10, 11). The immune complexes act as an antigen to stimulate B cells by activating the recognition receptors, such as Toll-like receptors (TLRs). TLR7 and TLR9 are the key receptors for the recognition of self-DNA or immune complexes and trigger the production of IFN-1 and inflammatory responses (12, 13). There are also some intracellular sensors in B cells and in other immune cells that can identify foreign DNA or self-DNA, including TLR9 and cyclic guanosine monophosphate–adenosine monophosphate, contributing to the overproduction of IFN-1 (14–16). Multiple signaling pathways are involved in the IFN-1 production process, which are activated after the interaction between sensors and DNA or another nucleic acid, such as pathways dependent on TLR9 and on stimulator of interferon genes (16, 17). As a key factor in the regulation of the innate immune response, IFN-1 can upregulate B cell-activating factor, promote B-cell differentiation, and suppress regulatory T cell (Treg) function (18, 19). With the help of IFN-1, autoreactive B cells undergo amplification, somatic mutation of immunoglobulin variable region genes, and class switch recombination, resulting in high-affinity immunoglobulin G (IgG) (20). In addition to suppressing Treg cells, IFN-1 can promote Th17 differentiation and increase the number of activated T cells (21). The insufficiency of Treg function contributes to the loss of immune tolerance in SLE via the TLR pathway (22). TLR9 can recognize dsDNA with a CpG motif and is dynamically upregulated in B cells (23). After a knockdown of TLR9, B cells produce fewer anti-dsDNA antibodies, and SLE syndrome is ameliorated in mice (24).

Moreover, autophagy is associated with the immune regulation and is essential for the homeostasis in immune cells. LC3-associated phagocytosis is an autophagy pathway that participates in the endocytosis of DNA or immune complexes by immune cells, especially plasmacytoid DCs. LC3 induces production of IFN-α via the TLR9 pathway in plasmacytoid DCs, whereas beclin-1, another member of the autophagy pathway, inhibits the production of IFN-α (25, 26). Beclin-1 knockdown macrophages can remarkably reduce antibody production and deposition of renal immune complexes, suggesting that autophagy may be an additional mechanism for the production of these antibodies (27).

Anti-dsDNA antibodies recognize diverse DNA structures, including single-stranded (ss)DNA, Z-DNA, bent or elongated dsDNA, DNA:RNA hybrids, locked-nucleic acids, and peptide: nucleic acid hybrids (28). Anti-dsDNA antibodies can bind to a complex of native dsDNA or modified DNA that contains a thymine dimer (generated during DNA photodamage). The chemically modified dsDNA may acquire a higher affinity for autoantibodies and form more stable complexes (29). Otherwise, antibodies can react with many non-DNA antigens in target cells and tissues: annexin II, α-actinin, laminin, collagen III, collagen IV, entactin, complement receptor type 1 (C1q), N-methyl-d-aspartate receptor (NMDAR), ribosomal P proteins, heparan sulfate, and others (30–39) (Table 1). Although there are no obvious similarities between DNA and potential cross-reactive antigens, some peptides can bind to both murine and human anti-dsDNA antibodies by mimicking a molecular DNA structure (2, 3).

**Table 1:** Antigenic recognition of anti-dsDNA antibodies.

**Self-antigen**	**Cells/Tissue**	**Contribution to SLE**	**References**
Annexin II	•Mesangial cells •Epithelial cells	•Activate p38 MAPK, JNK, and AKT •Induce secretion of IL-6	(30)
Alpha-actinin	•Mesangial cells	•Change cell shape and migration •Increase production of anti-chromatin antibody •Induce glomerular IgG deposition	(38)
Laminin	•Glomerular matrix	•Trigger inflammation •Damage the structure of ECM •Inhibit formation of the capillary structure	(40)
Collagen III/ IV	•Glomerular basement membrane •Glomerular matrix •Keratinocytes	•Exacerbate inflammatory infiltration •Activate the Fn14 and SOCS1 pathways	(33, 41, 42)
C1q	•Sera	•Induce immune complex deposition •Activate FcR-bearing cells •Induce production of anti-C1q antibodies	(36)
NMDAR	•Neuronal cells	•Apoptosis of neuronal cells •Enhance expression of proinflammatory factor •Damage blood-brain barrier	(43–45)
Entactin	•Glomerular basement membrane	•Increase production of anti-entactin antibodies •Promote the damage to glomerular basement membrane structure	(39)
Ribosomal P	•Hepatocytes •Lymphocytes •Mesangial cells	•Induce production of anti-Rib-P antibody •Enhance secretion of IFN-α and IL-10 •Induce T helper 1 responses	(37)
Heparan sulfate	•Glomerular basement membrane •Mesangial matrix	•Mediate penetration of living cells •Induce cell apoptosis and inflammation	(35, 46)

Anti-dsDNA antibodies have different subclasses, including IgA, IgE, IgG, and IgM. Nonetheless, not all of them contribute equally to tissue injuries in SLE. It is IgG and IgA but not IgM that correlate with disease activity in humans. Most pathogenic antibodies are affiliated with the IgG class in SLE patients (47). IgM antibodies seem to be protective by enhancing the elimination of apoptotic material and via immunomodulatory effects, thereby alleviating renal dysfunction (48). The class switch and somatic mutations of the IgG variable chain can increase the risk of pathological changes. Activation-induced deaminase (AID) in B cells is a key enzyme required for such processes, as confirmed in transgenic or non-transgenic murine models, where a loss of tolerance is accompanied by high production of class-switched IgG (49–51). When lacking AID, lupus-prone mice produce more anti-dsDNA IgM, but not the high-affinity IgG, and show substantial attenuation of glomerulonephritis and longer survival than do the wild-type mice (52). In SLE, DNA emerges as an inducer of an antigen-driven selection of B cells and is essential for a somatic mutation. During this process, the insertion of positively charged amino acids (for example, asparagine, arginine, and lysine residues) into complementarity-determining regions (CDRs) is critical for the binding of high-affinity dsDNA to the negatively charged phosphodiester backbone (53, 54). Among almost all IgG antibodies, anti-DNA IgG may have the potential for recognizing somatically produced idiotypes (53). Besides, the IgA and IgE subclasses of anti-DNA antibodies are implicated, independently or in combination with IgG, in lupus nephritis (LN) (47, 55, 56).

## The Detection of Anti-dsDNA Antibodies

Tests for anti-DNA antibodies can be positive at least 2 years before clinical diagnosis of SLE, and a surge in serum levels can present as a predictor for a severe SLE flare-up in the following 6 months (57). Subsequently, patients may undergo a course of relapsing–remitting with differences in the possibility of a flare between individuals. Anti-dsDNA antibodies emerge as a heterogeneous population in SLE, whereas they can be detected in some non-SLE patients with rheumatic symptoms, indicating that the employment of anti-dsDNA antibodies as a criterion for diagnosis and classification without subdivision may result in misdiagnosis and misclassifying of SLE (58, 59).

There are a variety of tests for anti-dsDNA antibody detection, such as the Farr radioimmunoassay (FARR-RIA), Crithidia luciliae indirect immunofluorescence test (CLIFT), and enzyme-linked immunosorbent assays (ELISA) (Table 2). The former two assays have been well-demonstrated for diagnosis and prognosis, whereas ELISAs are more valuable for detecting high-avidity anti-dsDNA antibodies in clinical laboratories and most closely associated with SLE activity (60). However, the materials used in assays limit their utilization, and researchers have been working on developing new suitable assays with high sensitivity and specificity. Panza et al. explored a novel assay that uses complexed histone peptides (PK201/CAT plasmid) with a fragment from Crithidia luciliae, which has more simple procedures (61). We utilized trypoanosoma equiperdum (TE) that containing uniformed dsDNA but no histone in the cell nucleus or kinetonucleus, which is much easier for purification with a simpler structure (62). Poulsen et al. developed a flow-induced dispersion analysis, offering a more sequence-specific antibody characterization via utilizing short and well-defined dsDNA sequences (63). It also exhibits the advantages of shorter analysis time and less sample volume consumption. Although the association between high levels of anti-DNA antibodies and disease activity has been widely appreciated, no signal-detecting approach can reliably evaluate disease activity in SLE. The current assays are not sufficient for detecting all of the antibodies in sera, especially the low-level antibodies, or the immune complex. Thus, at least two assays should be used for better evaluation and higher accuracy.

**Table 2:** Detection methods with strengths and weaknesses.

**Methods**	**Strengths**	**Weaknesses**
FARR-RIA	•Detect high-avidity antibodies •High specificity •Qualitative assay	•Low sensitivity •Use radioactive material
CLIFT	•Detect high-avidity antibodies •Qualitative assay •High sensitivity •High specificity	•Only score the kinetoplast fluorescence since nuclei always contain many antigens other than DNA •Lack of accurate serum titer
ELISA	•Detect low and high avidity antibodies •Quantitative assays •High sensitivity	•Low specificity •False-positive results due to binding of immune complexes (with negatively charged moieties)

## The Pathogenicity of Anti-dsDNA Antibodies

### Anti-dsDNA IgG Correlates Closely With LN

Among the affected organs, renal involvement indicates major internal damage in SLE patients. Anti-dsDNA antibodies are present in serum in nearly 80% of patients with LN. Anti-dsDNA antibodies directly or indirectly interact with renal antigens, thus producing immune complexes (32) Nevertheless, renal damage is not initiated solely by the complexes of a chromatin fragment and IgG depositing in the mesangial matrix or glomerular basement membranes. In some studies, enrichment of anti-dsDNA antibodies was not present in all extracted samples from the patients with proliferative LN and accounted for <10% of all antibodies, where overexpression of antibodies to C1q, Sm, SSA, and SSB was also responsible for the process (64). Similarly, researchers also discovered that a mouse strain (NZM.C57Lc4) with genetic modification has a severe renal disorder and the serological testing for anti-dsDNA antibodies yielded negative results (65). Moreover, anti-dsDNA IgG does not exert nephritogenic effects without the exposure of chromatin fragments in glomerular membranes and matrices (66). These results confirm that the initiation of LN is promoted by multiple autoantibodies, not by their single type. Reasonable factors that can explain this discrepancy including the subtypes of antibodies with differences in reactive specificity and affinity that have been described previously, differences in mouse strains utilized for the creation of animal models and other factors need to be illustrated in the future. Despite the doubts about their nephritogenic role in launching LN, the pivotal role of anti-dsDNA IgG and immune complexes in the acceleration of the LN process has been underscored by a wealth of evidence, where their effects on renal resident cells are well-documented (Table 3).

**Table 3:** Effects on renal cell types.

**Cell type**	**Proliferation**	**Inflammation**	**Fibrogenesis**
Mesangial cells	Increase/Decrease?	•↑ IL-1β, IL-8, IL-6, TNF-α, hyaluronan, lipocalin-2, iNOS, MCP-1	•↑ PKC, TGF-β1/MAPK, JAK2/STAT1 •↑ TGF-β1, F-actin •↓ SOCS1
PTECs	Increase	•↑ IL-6, IL-8, TNF-α, MCP-1, (NF)-κB, IP-10, MIP-1a, ICAM-1, VCAM-1	•↑ TWEAK/Fn14 •↑ MAPK, EMT, c-JNK, ERK, p38, Akt, JAK2/STAT1 •↓ SOCS1
Endothelial cells	Increase/Decrease?	•↑ IL-1β, IL-8, IL-6, TNF-α	•↑ JAK2/STAT1 ↓ SOCS1

Most immunoglobulins are unable to penetrate into living cells (67). However, anti-dsDNA antibodies can penetrate into cells and engage in an interplay with targets. Glomerular cells, hepatocytes, monocytes, fibroblasts, and neuronal cells are vulnerable to penetration by anti-DNA antibodies, mirroring the findings in the liver, spleen, and skin after treatment with penetrating antibodies *in vivo* (68, 69). The cellular penetration of antibodies is assisted by F(ab)′2 fragments with the mediation of the antigen-antibody binding region, which is also temperature-dependent and energy-consuming (70). Although the Fc fragment of an anti-DNA antibody contributes to its binding activity with monogamous bivalency, in which both Fab combining sites come into contact with DNA, the binding is not inhibited by the blockage of Fc receptor in mesangial cells (71, 72). In addition, the existence of manifold charged amino acids in CDRs is a specific feature of penetrating antibodies, which is explained by the energy-independent electrostatic interactions of arginine residues in the CDR2 and CDR3 with the negatively charged sulfated polysaccharides on the cell surface (68, 73), suggesting that cell penetration is an intrinsic property of anti-dsDNA antibodies. The DNA–histone complexes binding to purified cell-penetrating antibodies extracellularly can significantly enhance their subsequent cell entry depending on the environment (70). Besides, a wide variety of cognate nuclear antigens can function as endocytic receptors and promote subsequent internalization of antibodies into the cell, e.g., ribosomal P proteins (37), C1q (36), annexin II (30), α-actinin (38), and heparan sulfate (35, 46), further confirmed that the antigen-antibody mediation is crucial for the penetration mechanism. Although it is still unclear how anti-dsDNA antibodies actually cross the cell membrane, these data provide novel insights into antibody-binding endocytic receptors and elucidate their pathogenic role in SLE.

The penetrating anti-DNA antibodies can affect the proliferation and apoptosis of resident cells in LN, but the exact effect remains controversial. The anti-DNA IgG purified from the serum of LN patients has been shown to downregulate miR-10a and to trigger the proliferation of mesangial cells by targeting HOXA1, KLF4, and CREB1. Concomitantly, the expression of IL-6 is also enhanced, which is a crucial cytokine for promotion of the processes of cell proliferation and inflammation (74). Similar effects on cell proliferation are also seen in proximal renal tubular epithelial cells (PTECs) after exposure to the human anti-DNA antibodies; this treatment is associated with upregulated secretion of TNF-α, IL-1β, and IL-6 (75). These findings indicate that such antibodies can promote the proliferation of renal cells mainly by modulating the relevant inflammatory cytokines. These findings are also supported by the characteristic biopsy lesions of LN with deposition of immune complexes, hypercellularity of mesangial cells and epithelial cells, and infiltration by inflammatory cells. However, monoclonal and polyclonal anti-dsDNA antibodies show the ability to induce apoptosis, either by reacting with antigens on the cellular membrane or by entering the cell (76). Apoptosis can also be induced by these autoantibodies via upregulation of extracellular regulated kinase (ERK) activation and Bcl-2 production in murine mesangial cells. Endoplasmic-reticulum stress is upregulated in human mesangial cells after stimulation by anti-dsDNA antibodies, with activation of nuclear factor-kappa B (NF-κB) and increased expression of relevant inflammatory cytokines (72). This phenomenon may account for the apoptosis of mesangial cells, because enhanced endoplasmic-reticulum stress can cause cell death by changing the expression of multiple factors, including CHOP and Bcl-2, as discussed by Iurlaro (77). This discrepancy may be a result of the differences in the methods or circumstances of *in vitro* studies, but the diffuse proliferation can be observed in renal biopsy specimens from LN patients. Upregulation of apoptosis can manifest itself as DNA cleavage in inflammatory lesions as well as increased lysis of renal proximal tubular cells in the presence of complement (78). The apoptosis of podocytes leads to the destruction of glomerular basement membranes, and this event is accompanied by the aggravated progression of inflammation and fibrogenesis in renal tissues with the proliferation of mesangial cells and PTECs.

It is well-known that accumulation of inflammatory cytokines is sufficient for accelerating the recruitment of immune cells and the induction of inflammatory and fibrogenic processes promoting kidney damage. A wealth of proinflammatory factors, including monocyte chemotactic protein 1 (MCP-1), TNF-α, IL-1β, IL-6, IL-8, hyaluronan, lipocalin-2, and inducible nitric oxide synthase, are overexpressed in both human and murine mesangial cells upon stimulation with anti-dsDNA antibodies (72, 79, 80). Consistently with this evidence, the amounts of these cytokines have been shown to increase in human PTECs under the influence of anti-dsDNA antibody-mediated activation of the mitogen-activated protein kinase (MAPK) pathway; these factors further trigger the infiltration of monocytes and macrophages (81). These cytokines not only augment the infiltration by inflammatory cells but also increase the synthesis of fibronectin, eventually reproducing the features of tubulointerstitial fibrosis (41). Anti-dsDNA antibodies also sustain endoplasmic-reticulum stress and activated NF-κB accompanied by overexpression of cytokines in mesangial cells, resulting in a chronic inflammatory response and renal tissue damage (72). The high-titer anti-dsDNA antibodies are always accompanied by overexpression of TLR9 in podocytes, leading to increased production of TNF-α, IL-6, and IFN-γ (82). Besides, a multitude of signaling pathways are activated or involved in the autoantibody-mediated proinflammatory response. Excessive or persistent activation of tumor necrosis factor-like weak inducer of apoptosis (TWEAK)/fibroblast growth factor-inducible 14 (Fn14) signaling cascade positively correlates with the progression of LN. Anti-DNA IgG enhances the expression of Fn14 in keratinocytes through recognizing and binding to self-antigen, such as collagen III, indicating a relation between anti-DNA antibodies and the TWEAK/Fn14 signals (33). The mRNA expression levels of TWEAK and Fn14 increase in the glomeruli and tubulointerstitium in both LN patients and in a mouse model and act as an inducer of constitutive nuclear factor (NF-κB) activation, which mediates the expression of MCP-1, interferon γ-induced protein 10 (IP-10), macrophage inflammatory protein 1a, intercellular adhesion molecule 1, and vascular cell adhesion molecule 1 (83, 84). Additionally, MAPK, epithelial-to-mesenchymal transition (EMT), c-Jun N-terminal kinase, ERK, p38, and serine/threonine kinase Akt are also involved in TWEAK/Fn14 activation and downstream inflammatory and fibrotic responses (85, 86). Although the precise mechanisms of interaction of anti-DNA IgG and TWEAK/Fn14 have not been directly confirmed, anti-TWEAK antibodies, as well as Fn14 deficiency, are known to ameliorate renal damage induced by anti-dsDNA IgG in a murine model. Inhibition of TWEAK/Fn14 decreases renal IgG deposition without influencing serum anti-DNA IgG levels by relieving inflammation and protecting the filtration barrier, further confirming the crucial participation of the TWEAK/Fn14 signaling pathway in anti-DNA IgG-mediated LN (87–89).

**Figure 1:**
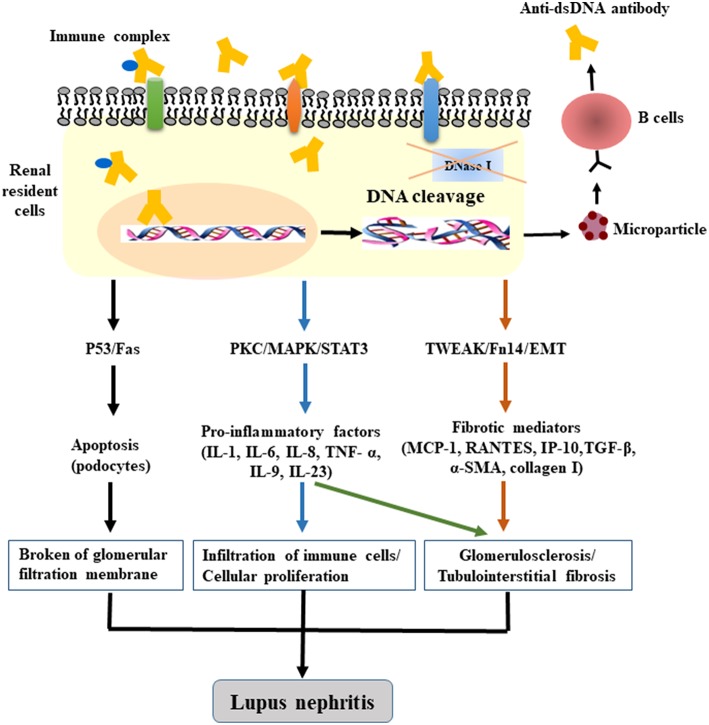
The pathogenic mechanism of anti-dsDNA antibodies in LN. After binding to DNA and non-DNA antigens, the penetrating anti-dsDNA antibodies relocate to the cytosol and cell nucleus, cause DNA fragmentation (accompanied by dysfunction of DNase), and induce apoptosis by regulating the gene expression of p53, Fas, or c-myc. The internalized anti-dsDNA antibodies enhance the expression of IL-6, IL-1β, TNF-α, and TGF-β1, activate the PKC, MAPK, TWEAK/Fn14, and EMT signaling pathways, and trigger the fibrotic process. Local deposition of anti-dsDNA IgG—in combination with the secretion of inflammatory or profibrogenic cytokines as well as the recruitment of immune cells—is sufficient for the initiation of renal fibrosis in LN.

A persistent chronic inflammatory response is always followed by fibrogenesis when there is no effective intervention, thereby leading to abnormal structure and dysfunction of kidneys with characteristic oversecretion of profibrogenic chemokines. Fibronectin is critical for fibrotic progression in glomerulonephritis and is distributed widely in the mesangial matrix, glomerular, and tubular basement membranes, and Bowman's capsule. The formation of glomerular fibronectin is enhanced both in patients and mice with active LN (90, 91). We have demonstrated that anti-dsDNA IgG contributes to renal fibrosis by downregulating suppressor of cytokine signaling 1 (SOCS1) and activating Janus kinase/signal transducer and activator of transcription 1 signals, which modulate the expression of profibrotic genes, transforming growth factor beta 1 (TGF-β1), platelet-derived growth factor B, and connective tissue growth factor (92, 93). In addition to SOCS1, protein kinase C signaling is also simulated by anti-dsDNA antibodies with increased secretion of TGF-β1 (94). As a profibrotic growth factor, TGF-β1 is responsible for the cellular myofibroblast-like phenotype switch, in which the assembly of the actin cytoskeleton induces the synthesis of plasminogen activator inhibitor 1 through the TGF-β1/MAPK pathway in mesangial cells, with an accumulation of the extracellular matrix (95–97). Moreover, pathogenic anti-dsDNA antibodies cross-react with alpha-actinin 4; these data account for the promotion of myofibroblast-like phenotypic changes via upregulation of fibrogenic factors in mesangial cells, including F-actin and TGF-β1, as confirmed both *in vitro* and *in vivo* (38, 98). Anti-DNA antibodies can also enhance the phenotypic changes or EMT in cultured PTECs. The activation of EMT with increased mesenchymal markers contributes to the interstitial infiltration by leukocytes and to tubule-interstitial fibrosis (75). Therefore, we can conclude that anti-dsDNA antibodies play a crucial part in the inflammatory and fibrogenic mechanisms of LN (Figure 1); however, the complicated network of inflammatory cytokines and intricate signaling pathways should be investigated further.

### Anti-dsDNA IgG Participates in Other Injuries

Skin is frequently affected by SLE and can be the only affected organ. The serum positivity for an anti-dsDNA antibody is observed in only a subgroup of patients with cutaneous lupus erythematosus. The binding specificity of anti-DNA IgG to skin sections and keratinocytes has been observed *in vitro*, which can be promoted by ultraviolet light irradiation, resulting in keratinocyte apoptosis via antibody-dependent and cell-mediated cytotoxicity (33, 99). The deposition of an immune complex at the dermoepidermal junction has been confirmed in both murine models and patients with serological positivity for anti-DNA IgG (33). Actually, the mild inflammatory response in combination with the presence of autoantibodies is necessary for further cross-reactions with collagen III, collagen IV, and SOCS-1 and−8 for induction of antibody accumulation in skin tissue (42, 100). In addition to LN, the role of TWEAK/Fn14 signals in cutaneous lupus erythematosus has been discovered in murine lupus models (87, 101–103). Ultraviolet B irradiation promotes the anti-DNA IgG binding to keratinocytes as well as the expression of Fn14, which engages in an interplay with TWEAK and subsequently enriches the expression of proinflammatory factors and induces apoptosis (33, 103). These induced factors, including IL-6, IL-8, MCP-1, and RANTES (regulated upon activation, normal T cell expressed and secreted), further amplify the inflammatory responses by attracting immune cells (101).

Central nervous system (CNS) diseases are associated with a poor prognosis among patients with SLE and have been increasingly reported during the past decade. Neuropsychiatric complications occur in 30–40% of patients with SLE and may constitute the initial presentation or a flare (89). Anti-phospholipid, anti-N-methyl-D-aspartate receptor (NMDAR), anti-ribosomal-P, and anti-dsDNA antibodies account for the progression of CNS disease (43). Anti-dsDNA antibodies can cross-react with NMDAR on neurons. An extracellular epitope has been identified near amino acid residue 369 of GlyN1 in NMDAR and decreases the density of the surface receptor, causing further neuronal disorders, such as abnormalities of cognition and memory (43, 44). Enriched serum anti-dsDNA and anti-NMDAR antibodies are associated with the emergence of neuropsychiatric lupus symptoms (45). Nevertheless, an increased titer of an anti-DNA antibody is not always consistent with neurological dysfunctions (104). The inconsistency may be due to the differences in cognitive assessments applied in diverse studies and the samples tested. The anti-NMDAR/dsDNA antibodies can generate and gain access to the brain tissue after the destruction of the blood-brain barrier, leading to neuronal cell death and abnormalities in mice (45). Injection of mouse brains with an anti-DNA antibody (R4A) causes apoptosis of hippocampal cells (105). Besides, we observed enhanced expression of proinflammatory factors as well as abnormal cognition and behavior in wild-type mice, while the cognition and integrity of the blood-brain barrier are attenuated in Fn14-deficient mice, indicating the amplification of TWEAK/Fn14 signals in the pathogenesis of CNS diseases (106).

### The Constant Region Contributes to the Antigenic Specificity and Pathogenicity of Anti-DNA Antibodies

Classically, the variable region is the only part of an antibody that is credited with the antigenic specificity, whereas the functions of antibodies are determined by the constant region. These two domains are believed to be structurally and functionally independent. However, the specificity and affinity of antibodies are believed to be determined by both regions but not solely by the variable one (107). The variety of isotypes of anti-DNA antibodies is generated through the isotype switch recombination in the sequence from IgM to IgG, whose variable regions are identical to those of the initial antibody. Particular subclasses of anti-DNA antibodies are more closely associated with a pathogenic potential (47). In lupus patients, serum anti-DNA IgG1 is always elevated before the occurrence of renal relapse, while IgG2a, IgG2b, and IgG3 are more frequently eluted from kidneys with active nephritis (78, 108). In addition, different IgG subclasses with similar specificity for DNA show a remarkable difference in the binding properties for basement membranes, the formation of immune-complex deposits, and the severity of the induced proteinuria (109).

To validate the association between subclasses and pathogenicity, we found that different isotypes of anti-dsDNA antibodies differ from each other in the recognition specificity of nuclear and renal antigens when we generated a panel of murine anti-DNA antibodies (IgM, IgG1, IgG2a, IgG2b, and IgG3), which contain identical variable-joining-diversity (VDJ) regions, suggesting that the effect of the constant region on antibody binding is directly associated with autoimmunity (34). In fact, the factors responsible for the pathogenicity of anti-DNA antibodies are diverse, including their binding avidity for DNA, cross-reactivity for self-antigens, characteristics of the idiotype, and localization of specific amino acid residues in CDRs (110). Additionally, the diverse subclasses of autoantibodies that determine the complement fixation and Fc binding are crucial in LN. The isotypes that form the immune complex can fix complement—in a process involving IgG1 and IgG3 in humans and IgG2a and IgG2b in mice—upregulated in the renal glomerulus (111, 112). IgG2a and IgG2b manifest the most robust complement activity and binding affinity for triggering Fc receptors III and IV and are the most potent autoantibodies during the mediation of hemolytic anemia *in vivo* (108). Although IgG2a is the predominant subclass among IgG-reactive IgGs and is present as the most pathological subclass in murine lupus models (113), IgG3 derived from MRL/lpr mice is crucial for the spontaneous glomerular disruption and dysfunction (109). Besides, the constant region of antibodies affects their binding ability toward DNA and histones not only by varying the environments of their paratopes but also by altering their secondary structures (34, 114). Nevertheless, the prognostic value of these subclasses of anti-dsDNA antibodies remains to be illustrated.

### The Catalytic Properties of Anti-dsDNA IgG

**Figure 2:**
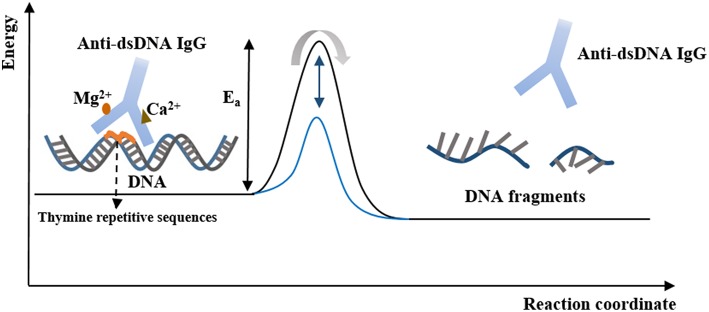
The catalytic properties of anti-dsDNA IgG. Anti-dsDNA IgG binds to DNA at the thymine repetitive sequences via tyrosine side chains within a hydrophobic pocket. Hydrolysis of DNA is an energy-intensive process and can be activated by the binding of Ca^2+^ and Mg^2+^. After binding to DNA, the active site of IgG is converted to a transition state, and the DNA fragments are produced and released. At this point, the free IgG binds to another DNA molecule and begins its new cycle, in which IgG stabilizes the transition state of the reaction and lowers the activation energy, and thereby increases the rate of the reaction.

The first IgG antibody with DNA-related catalytic activity in SLE was found in 1992 (115). Antibodies binding to nucleoprotein complexes, to DNA, and enzymes that account for nucleic acid metabolism may acquire the feature of the primary antigen-catalytic activity. The amino acid residues of catalytic anti-dsDNA antibodies and DNA enzymes may have similarities because anti-dsDNA antibodies in patients with SLE have been shown to hydrolyze plasmid DNA (116–118). The catalytic activity of anti-DNA antibodies is associated with the binding of magnesium and calcium ions, and their cytotoxicity results from the hydrolysis of nuclear matrix proteins (Figure 2) (116). Moreover, the variety of light chains of anti-DNA IgG, the tolerance of temperature, optimum pH, and hydrolysis rate are thought to be responsible for distinct activities of anti-DNA antibodies and their capacity for DNA hydrolysis (119).

In the sequence analysis of anti-DNA antibodies from humans and mice, there is a high frequency of somatic mutations in the VH and VL sequences of anti-dsDNA IgG with high affinity (53). Such somatic mutations result in a higher proportion of certain amino acids, especially arginine, asparagine, lysine, and tyrosine in the CDRs; this phenomenon promotes the formation of electrostatic interactions and hydrogen bonds between these amino acids and DNA (53). In our study, anti-dsDNA antibodies of various isotypes (PL9-11 clone) that share identical variable regions but different constant regions could cleave not only dsDNA but also peptide antigens depending on the isotype, e.g., the ALWPPNLHAWVP peptide, indicating that the catalytic cleavage of DNA can be regarded as a natural property of these antibodies (120). The specific affinity of anti-DNA antibodies to antigens probably determines the possibility of catalysis, but not the catalytic efficiency of the reaction. Although antibodies derived from various sources contain similar variable-chain sequences, these antibodies have different catalytic activities. In addition, in the context of this specific autoantibody system, a specific mutation results in the reduction of DNA binding as well as weakened catalytic activity (121). The DNA binding and hydrolytic activities of anti-DNA antibodies are well-conserved in both VH and VL. The catalytic characteristics of anti-DNA antibodies are isotype-dependent, and constant regions have an influence on the autoantibody-antigen interaction presumably by modifying the secondary structure of antibodies (34, 114, 120).

## The Blockade of Pathogenic Anti-dsDNA IgG

### An iDC Vaccine Suppresses the Production of Anti-dsDNA IgG

This therapeutic strategy against SLE is focused on eliminating or controlling active B cells that offer limited benefits but hamper the advancement of treatment for SLE (122, 123). Recently, the significance of iDCs in the induction of clonal anergy and immune tolerance was highlighted. iDCs participate in the anergy of T cells by inducing the apoptosis and promoting the differentiation of Treg cells because the expression of costimulatory factors and MHC molecules on the surface of iDCs decrease considerably (124). As a key cytokine for inhibiting excessive immune responses, IL-10 can reduce the production of relevant cytokines (such as TNF-α and IL-1β,) as well as increase production of inhibitory factors (such as programmed death ligand 1) in DCs, thereby inhibiting Treg responses and the function of effector T cells (125–127). Among these factors, programmed death ligand 1 in combination with TGF-β is particularly important for promoting the conversion of CD4+ T cells to Foxp3+ CD4+ Treg cells, whereas blockade of programmed death ligand 1 can reduce anti-dsDNA IgG production and immune-complex formation in lupus-prone mice by suppressing the synthesis of IL-10 in CD4+ Treg and B cells, thus further validating the therapeutic potential of iDCs (128, 129). After the induction of clonal anergy, T cells fail to bind to cognate antigens that are presented by DCs.

The iDCs exert weaker activating effects on T cells by expressing lower amounts of costimulatory cytokines, including IL-6 and IL-12 in patients with SLE; this observation is consistent with iDCs' effects in lupus-like mice where iDCs lower the responses of Th1/Th2 cells and thus inhibit the secretion of IL-2, IL-4, IL-12, and interferon γ and the formation of anti-dsDNA IgG (130–132). Besides, iDCs from hemopoietic stem cells can be loaded with nuclear antigens, thereby serving as a live cell vaccine (131, 132). Thus, iDCs are emerging as promising immunomodulators in SLE. Nonetheless, there are many challenges regarding the appropriate production protocols and administration route and timing of an iDC vaccine. Moreover, the exact mechanism by which iDCs interact with Treg cells, B cells, and other cells, as well as the possible side effects are largely unclear. Therefore, the translational application of the iDC vaccine in clinical settings requires multidisciplinary efforts.

### DNA-Mimicking Peptides Block the Binding of Anti-dsDNA IgG

Based on the interactions between self-antigens and anti-dsDNA antibodies, novel therapeutic peptides that bind to autoantibodies have attracted increasing attention. These peptides can be synthesized by combining desired amino acid residues or by chemical modification of certain sequences, to ensure higher activity and specificity. To date, therapeutic peptides have proven to be effective against experimental autoimmune diseases, for example, rheumatoid arthritis and multiple sclerosis (133). Moreover, some therapeutic peptides have been demonstrated to prevent anti-dsDNA antibodies from binding and reacting with target antigens and tissues in the studies on SLE.

Peptide DWEYS (D/E W D/E Y S/G), a part of NMDAR expressed on neurons, has been shown to bind to anti-dsDNA antibodies, thereby disrupting the neurotoxicity of anti-DNA antibodies through the NMDAR-activated pathway, in which circulating antibodies penetrate the hippocampal pyramidal cells followed by impairment of spatial cognition (134). In addition, peptide DWEYS can prevent the proliferation of both B cells and T cells and ameliorate the production of anti-DNA IgG and proteinuria in lupus mice by selectively suppressing the autoreactive B lymphocytes via cross-reaction with the immunoglobulin receptors on B cells (135). Although the DWEYS peptide is certainly beneficial for therapeutic applications owing to the small molecular weight and non-immunogenicity, it is not highly useful due to its short half-life.

FISLE-412, an analog of a reduced protease inhibitor for the human immunodeficiency virus, has the potential to block DNA recognition of anti-dsDNA antibodies (136). FISLE-412 can reduce glomerular deposition of IgG, protect kidneys from damage, and suppress the onset of SLE in murine models. Moreover, the FISLE-12-bound anti-dsDNA antibodies exert no neurotoxic effects on C57BL/6 mice; this finding shows a greater neuroprotective effect than that of the DWEYS peptide (136). As compared with DWEYS, FISLE-412 has higher affinity and a more stable structure and is more suitable for oral administration (136). The study on the structure-activity relationship of several synthesized analogs of FISLE-412 has revealed that the hydrophobic portion is the key region for this therapeutic function (137). In addition, the quinaldic region contributes to the binding activity of FISLE-412 (137).

ASPVTARVLWKASHV is a 15-mer peptide selected by purified polyclonal anti-dsDNA IgG in lupus patients and can inhibit antigen-IgG binding (138). ALW (ALWPPNLHAWVP) is a 12-mer peptide mimic originating from a panel of murine anti-DNA IgG isotypes; it can bind to all four IgG isotypes and prevent IgG binding to DNA or glomerular antigens (3). ALW is physiologically stable and resistant to oxidation, cyclization, and degradation because it lacks a sequence of certain amino acid residues (3). Although ALW and its mimics are receiving increasing attention as therapeutic molecules with less toxicity for lupus patients, a combination of different peptides should be taken into consideration because of the variability and cross-reactivity of anti-DNA antibodies. In addition, peptides have low toxicity when administered orally or subcutaneously and would not be expected to be immunogenic.

pCons (FIEWNKLRFRQGLEW) is a 15-mer peptide derived from murine anti-dsDNA antibodies; it exerts therapeutic effects by preventing antibody-antigen binding (139). pCons significantly enhances the survival rate and alleviates glomerulonephritis in NZB/W lupus mice; this beneficial action may be mediated by the regulation of T-cell autoreactivity (140, 141). The protective effects have been validated in lupus-prone mice treated with a B lymphocyte gene vaccine that codes for IgG1 Fc-pCons, where early and repeated administration of the vaccine delayed proteinuria and enhanced survival remarkably as well as the expansion of Foxp3+ CD4+ Treg cells (142).

hCDR1 (GYYWSWIRQPPGKGEEWIG) is a 19-mer peptide that is based on CDR1 sequences from human anti-DNA antibodies. hCDR1 has suppressive effects on T cells by reducing apoptosis of T cells with less secretion of interferon-γ and upregulation of IL-10 in lupus-prone mice (143). The effects of hCDR1 on B cells are also associated with downregulated levels of anti-DNA antibodies in lupus-prone mice (144). In an early clinical study, weekly subcutaneous administration of different doses of hCDR1 (Edratide)-−0.5, 1.0, and 2.5 mg—was found to be effective in ameliorating the manifestations and in downregulating the production of proinflammatory and proapoptotic cytokines in SLE patients; the affected proapoptotic proteins include IL-1β, TNF-α, IFN-γ, IL-10, caspase 3, and caspase 8 (145). The efficacy of hCDR1 in the treatment of SLE was questioned because of the limited sample size (9 patients) and disease severity from mild to moderate among the patients. In a later clinical phase II trial, the injection of hCDR1 once a week at a dose of 0.5 mg in SLE patients with a larger sample size (340 patients) was found to be safe and effective, with a decreased SLE disease activity index (SLEDAI-2K). However, there was no remarkable difference between the hCDR1 group and placebo group. In addition, the shortest course of efficacy proven by this trial was nearly 1 year, and the primary endpoint was not reached; this factor may be responsible for the insignificance of the therapeutic effects (146). Thus, a longer-term and appropriate endpoint should be scheduled in later studies on hCDR1.

## Main Conclusions and Further Investigations

Anti-dsDNA antibodies, the hallmark of SLE, constitute a potent parameter for classifying and diagnosing SLE. Their contribution to damage to the kidneys, skin, and brain in SLE has been well-documented. The production of anti-dsDNA antibodies results from combining multiple factors, including abnormalities of dendritic cells, B cells, or T cells and deficiency of a DNase that leads to failure of cleaning released nuclear materials; however, it still need further studies. The effects of penetrating autoantibodies on triggering a complicated inflammatory and fibrotic process underlie the role of anti-DNA antibodies in damaging target cells and organs. The pathogenicity of antibodies highlights the promising therapeutic potential of DNA-mimicking peptides that react with these autoantibodies in SLE, which can ameliorate the manifestations of SLE in murine models. However, the clinical trials progress slowly in which the timing, dosages, route of administration, alteration of bioactivity, and possible adverse effects of such peptides should be taken into account for developing more efficient therapies for SLE.

## Author Contributions

YX conceived this paper. XW wrote this manuscript. XW and YX read and approved the final manuscript.

### Conflict of Interest Statement

The authors declare that the research was conducted in the absence of any commercial or financial relationships that could be construed as a potential conflict of interest.
